# Association between Preoperative Plasma sRAGE Levels and Recovery from Cardiac Surgery

**DOI:** 10.1155/2013/496031

**Published:** 2013-09-05

**Authors:** Benedict C. Creagh-Brown, Gregory J. Quinlan, Lauren R. Hector, Timothy W. Evans, Anne Burke-Gaffney

**Affiliations:** ^1^Unit of Critical Care, Respiratory Science, National Heart and Lung Institute Division (NHLI), Faculty of Medicine, Imperial College London, Dovehouse Street, London SW3 6LY, UK; ^2^NIHR Respiratory Disease Biomedical Research Unit, Royal Brompton and Harefield NHS Foundation Trust, London SW3 6NP, UK

## Abstract

*Background*. The receptor for advanced glycation end products (RAGE) is an inflammation-perpetuating receptor, and soluble RAGE (sRAGE) is a marker of cellular RAGE expression. This study investigated whether raised plasma levels prior to surgery of sRAGE or S100A8/A9 (a RAGE ligand) were associated with longer duration of hospital care in patients undergoing cardiac surgery necessitating cardiopulmonary bypass. *Methods*. Patients (*n* = 130) undergoing elective cardiac surgery were enrolled prospectively. Plasma sRAGE and S100A8/A9 concentrations were measured before and 2 h after surgery. *Results*. Preoperative plasma sRAGE increased significantly (*P* < 0.0001) from 1.06 ng/mL (IQR, 0.72–1.76) to 1.93 ng/mL (IQR, 1.14–2.63) 2 h postoperatively. Plasma S100A8/9 was also significantly (*P* < 0.0001) higher 2 h postoperatively (2.37 **μ**g/mL, IQR, 1.81–3.05) compared to pre-operative levels (0.41 **μ**g/mL, IQR, 0.2–0.65). Preoperative sRAGE, but not S100A8/A9, was positively and significantly correlated with duration of critical illness (*r* = 0.3, *P* = 0.0007) and length of hospital stay (LOS; *r* = 0.31, *P* < 0.0005). Multivariate binary logistic regression showed preoperative sRAGE to be, statistically, an independent predictor of greater than median duration of critical illness (odds ratio 16.6, *P* = 0.014) and to be, statistically, the strongest independent predictor of hospital LOS. *Conclusion*. Higher preoperative plasma sRAGE levels were associated with prolonged duration of care in adults undergoing cardiac surgery requiring cardiopulmonary bypass.

## 1. Introduction 

First described as a receptor (R) for advanced glycation end products (AGEs), RAGE is an immunoglobulin-like pattern recognition receptor constitutively expressed at low levels in all cells and at high levels in the lung [1, 2]. RAGE also interacts with a range of damage-associated molecular pattern molecules including the S100 proteins. S100A8/A9 is a heterodimeric S100 protein released from leukocytes during inflammation [3], and plasma, leukocyte, or bronchoalveolar lavage levels correlate with disease activity in patients with septic shock [4–6] or acute respiratory distress syndrome (ARDS) [7]. 

RAGE is considered to be an inflammation-perpetuating receptor because receptor activation leads to further RAGE expression and sustained *de novo* synthesis of the inflammatory transcription factor NF-*κ*B. Higher preoperative levels of plasma sRAGE could be associated with a worse outcome following surgery [8]. A “two-hit” model describes the role of RAGE in inflammation which hypothesizes that chronic conditions such as renal failure or diabetes and increasing age *per se* [9], some of the preoperative risks factors for cardiac surgery, represent a first inflammatory “hit,” leading to widespread upregulation of RAGE expression [1]. A second, superimposed acute inflammatory insult, such as cardiac surgery necessitating (sn) cardiopulmonary bypass (CPB) or other conditions associated with hyperglycaemia, ischaemic stress, and release of inflammatory mediators, leads to a further increase of RAGE ligands [1]. 

A soluble form of RAGE (sRAGE) also exists and consists of both shed membrane-bound RAGE ectodomain and released endogenous secretory (es) RAGE [10]. Shed and esRAGE are ligand-binding, have no signal transduction capacity, and are often detected in plasma as a single entity without distinction. Soluble RAGE is likely to act as a decoy molecule to bind excess RAGE ligand and increased levels could be considered protective [11]. However, it is also possible that the production of sRAGE is an attempt by cells to reduce cell surface receptor expression and to limit cellular activation, in which sRAGE levels can reflect the extent of cellular RAGE expression and activation. Whether levels of sRAGE are associated with better or worse outcome might, in part, reflect these two roles.

A number of studies have investigated associations between plasma levels of sRAGE levels and outcome in a number of critical illnesses [1, 12, 13]. An association between adverse outcome and high plasma sRAGE levels has been extensively studied in patients with ARDS [14–17]. Plasma sRAGE levels are also raised in patients with acute pancreatitis, with the highest levels in those with severe organ failure [18]. In patients with sepsis [19] and pneumonia [20], the highest levels were detected in nonsurvivors; and in trauma victims, levels were correlated with the development of acute renal failure [21]. In children undergoing cardiac surgery, sRAGE levels increased and correlated with outcome [22], and in adults, levels were raised postoperatively, but associations with outcome were not reported [23]. However, whether raised levels of sRAGE prior to a second inflammatory insult such as snCPB result in poorer outcome has not been investigated.

In order to test the hypothesis that RAGE axis activity might be a prognostic biomarker, we measured sRAGE and the sRAGE ligand, S100A8/A9, in the plasma of patients undergoing complex or high risk snCPB, and we investigated associations with clinical characteristics, operative values, and outcome variables. 

## 2. Methods

### 2.1. Patient Population and Sample Collection

Samples and clinical data were collected, prospectively, from patients at the Royal Brompton Hospital, London, UK (from 2007 to 2010), who were undergoing cardiac snCPB. Patients preferentially recruited were those requiring complex surgery (i.e., valve replacement surgery and coronary artery bypass grafting or more than one heart valve replaced/repaired) or, alternatively, those deemed high risk (i.e., additive EuroSCORE ≥ 6 [24]). These criteria were used to select patients with a greater likelihood of elevated preoperative sRAGE, a prerequisite for our hypothesis. The only exclusion criterion was lack of informed consent. Blood was collected via indwelling cannulae and if not for immediate analysis, it was processed and plasma samples stored at −80°C. Plasma samples were collected immediately prior to and 2 h following snCPB (interleukin-8 (IL-8), sRAGE, and S100A8/A9) or on the first postoperative day (C-reactive protein (CRP)).

### 2.2. sRAGE and S100A8/A9 Assays

Commercially available sandwich enzyme-linked immunosorbent assay (ELISA) kits were used to measure sRAGE (R&D Systems, Oxford, UK) and S100A8/9 (NovaTec Immundiagnostica GmbH, Germany). sRAGE was measured in 129 samples due to loss of one sample.

### 2.3. Clinical Data

Preoperative characteristics, age, gender, body mass index (BMI), creatinine, and additive EuroSCORE were recorded. Operative and postoperative variables were obtained following interrogation of an automated clinical data collection system. In patients for whom a sufficient sample was available, CRP (*n* = 106) and IL-8 (*n* = 122) were measured as markers indicative of systemic inflammation after snCPB using the N high-sensitivity CRP monoassay with the BN ProsPec Nephelometer (Dade Behring, Deerfield, Illinois) and a bead array assay (xMAP, Luminex, Texas, USA), respectively. CRP was selected as it is one of the most widely measured markers of inflammation; and IL-8 was selected, because extensive literature supports an association between this proinflammatory cytokine and adverse postoperative sequelae. Together, raised levels of these biomarkers are indicative of a second inflammatory “hit” after-surgery.

The measure of oxygenation employed was PaO_2_ : FiO_2_ ratio (PFR). The first value recorded on return from theatre to the intensive care unit (ICU) was used as a measure of pulmonary organ dysfunction or respiratory failure. Duration of mechanical ventilation and incidence of acute lung injury were recorded. Indices of clinical outcome included ICU, hospital lengths of stay (LOS), and mortality. ICU LOS was employed as a composite index reflecting the degree of physiological derangement and the requirements for supportive care. To obviate the influence of nonclinical considerations such as “step-down” bed availability, electronic databases of patient physiological parameters were analysed, and the duration that patients required advanced therapies and monitoring was determined, as recommended previously [25]. “Level 3 care” duration is one such calculated variable; derived from the UK Critical Care Minimum Data Set (CCMDS), it is defined as the time in hours that a patient requires advanced therapies and monitoring at a level that would be usually thought of as “intensive care” [26]. 

The study protocol was approved by the hospital research ethics committee, and informed consent was obtained from all patients. Reporting of the study conforms to STROBE and the broader EQUATOR guidelines [27].

### 2.4. Statistics

Statistical analyses were performed to establish associations between plasma biomarkers and clinical indices. Nonparametric data were expressed as median and interquartile range (IQR) and compared using Wilcoxon matched-pairs test with Dunn's correction for 2 paired groups and Friedman's test for 3 paired groups. Parametric data were expressed as mean ± standard error of the mean (SEM) and compared using a paired Student's *t*-test. Correlations between variables were assessed using Spearman analysis. *P* values of <0.05 were considered significant. Multivariate binary logistic regression was used to determine associations between patient characteristics and plasma biomarker values and, in particular, to assess independence of effects. Patient characteristics and assay values were, generally, parametric in distribution; those that were not were log_10_ transformed. As outcome is binary but duration of level 3 care is a continuous variable, duration of care was divided into quartiles and expressed as level 3 care > median (19.8 h). Data were expressed as odds ratio with 95% confidence interval. Excel 2007 (Microsoft, CA), Prism v5.04 (Graphpad software, CA), and SPSS v17.0 (IBM, CA) were used for analyses. Statistical advice was obtained from the Department of Medical Statistics, Royal Brompton and Harefield NHS Foundation Trust.

## 3. Results 

### 3.1. Patient Characteristics 

The demographics, clinical characteristics, operative variables, and postoperative outcomes of the patient population (*n* = 130, 62.3% men) are shown in Table 1. In comparison to the average patient undergoing snCPB in the UK, these patients were older and generally underwent more complex surgery yet survival was comparable to the national average [28]. Ischemic time of 85.5 min (IQR 67–108) was similar to mean aortic cross clamping time (85–87 min) reported in a recent study with patients of similar complexity [29]. Postoperative systemic inflammation was confirmed by significant elevations in plasma levels of IL-8 (*n* = 122) 2 h after snCPB and CRP (*n* = 106) measured postoperatively on day one (Table 1). Median duration of level 3 care required was 19.3 h (IQR 13.7–40.8), and hospital LOS was 8.9 days (IQR 7.2–13.1). Overall hospital mortality was 5%. 

### 3.2. Plasma Levels of sRAGE and S100A8/9 Increased after snCPB

**Figure 1:**
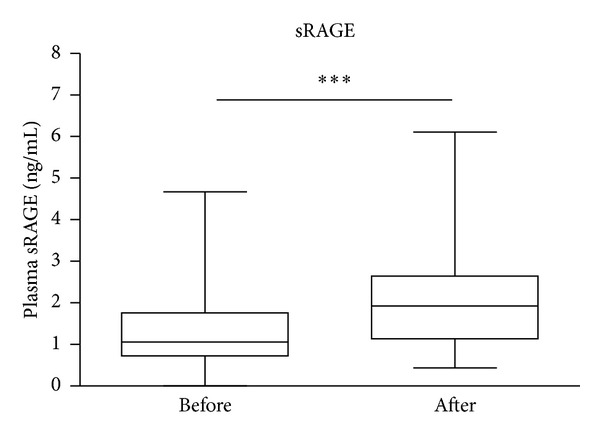
Plasma levels of sRAGE increased 2 h after surgery necessitating cardiopulmonary bypass compared with preoperative levels. Data presented as bar graphs with median and 75th percentile concentrations of sRAGE indicated *n* = 129, ***P < 0.001.

**Figure 2:**
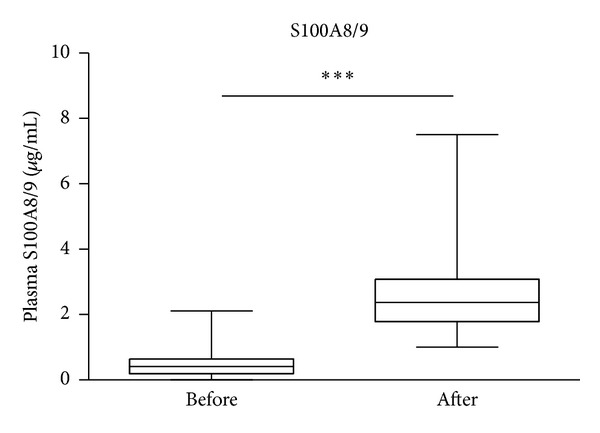
Plasma levels of S100A8/9 increased 2 h after surgery necessitating cardiopulmonary bypass compared with preoperative levels. Data presented as bar graphs with median and 75th percentile concentrations of S100A8/A9 indicated *n* = 130, ***P < 0.001.

Plasma levels of sRAGE were significantly (*P* < 0.0001, *n* = 129) higher in patients 2 h after snCPB (1.93 ng/mL, IQR 1.14–2.63) compared with levels in paired samples taken prior to surgery (1.06 ng/mL, IQR 0.72–1.76; Figure 1). Plasma levels of S100A8/9 were significantly (*P* < 0.0001, *n* = 130) higher in patients 2 h after snCPB (2.37 *μ*g/mL, IQR 1.81–3.05) compared with those in paired samples taken prior to surgery (0.41 *μ*g/mL, IQR 0.20–0.65; Figure 2). 

No significant correlations were found between postoperative levels of sRAGE and the patient characteristics, operative variables, or post-operative outcomes shown in Table 1, including PFR (*r* = −0.07, *P* = 0.4) or duration of ventilation (*r* = 0.19, *P* = 0.03). No difference was shown between sRAGE levels in those with PFR < 200 mmHg and those with >200 mmHg (defining cutoff for acute lung injury (ALI)). Moreover, there were no significant correlations between postoperative levels of S100A8/A9 with patient characteristics, operative variables, or postoperative outcomes except duration of CPB (*r* = 0.32, *P* < 0.0002). 

### 3.3. Preoperative sRAGE Level Is an Independent Determinant of Outcome

Whilst there were no associations between preoperative levels of S100A8/A9 and patient characteristics, operative variables, or postoperative outcomes shown in Table 1, significant positive associations were found between preoperative sRAGE levels with duration of level 3 care (*r* = 0.30, *P* = 0.0007) and hospital LOS (*r* = 0.31, *P* = 0.0005). 

Thus, duration of level 3 care was dichotomised into less and greater than median care, and binary logistic regression analysis was applied. Variables with statistically significant relationships to greater than median duration (19.25 h) were ischemic time, CPB time, log preop and postop sRAGE, and log postop S100A8/A9 (Table 2). Of these, the only variable with an independent relationship following multivariate analysis was log preop sRAGE (odds ratio 16.6, *P* = 0.014; Table 2). When a similar approach was applied to hospital LOS, greater than median stay was significantly associated with similar variables and also age. On multivariate analysis preop Log sRAGE and age remained statistically significant with preop sRAGE being the strongest predictor, statistically, of prolonged duration of hospital LOS (Table 3). 

## 4. Discussion 

This study has shown that plasma levels of sRAGE and S100A8/9 were raised postoperatively in patients after snCPB. However, increased levels did not correlate with patient characteristics, operative variables, or postoperative outcomes with the exception of an association between S100A8/A9 and duration of CPB. By contrast, preoperative sRAGE levels correlated, significantly, with prolonged duration of level 3 and hospital care. Moreover, preoperative sRAGE was the only variable, when duration was categorised as greater or less than the median, with an independent relationship to duration of level care 3 and, statistically, the strongest predictor of hospital LOS. These novel findings suggest that higher preoperative plasma sRAGE levels are indicative of longer recovery after cardiac surgery requiring CPB, suggesting increased RAGE activation prior to surgery is not advantageous. 

S100A8/A9 makes up 40% of the cytosolic protein in neutrophils, is released either through nonclassical secretion or through loss of membrane integrity during necrosis, and acts as a neutrophil chemoattractant and activator [30]. Our findings of increased levels of S100A8/A9 support previous reports of increased expression in other acute and chronic inflammatory conditions including sepsis and septic shock [4, 6, 31]. In particular, our findings correspond with a previous study showing increased S100A8/9 gene expression in circulating leukocytes following CPB [32]. That levels in our study correlated, positively, with duration of CPB suggests that injurious factors consequent on surgery together with CPB, including activation of blood during exposure to the extracorporeal circuit; relative tissue hypoperfusion, myocardial, and pulmonary ischemia; and operative tissue injury [33], trigger release of S100 proteins. A study in children undergoing cardiac surgery requiring CPB showed another leukocyte S100 protein, S100A12, increased postoperatively and levels were correlated with respiratory parameters, ICU and hospital length of stay [22]. However, the patient group in this study by Liu and colleagues was dissimilar to ours; not least, because a number of children in their study developed ALI, whereas no adult in our study group did. For this reason, we chose not to measure markers of lung injury such as surfactant derived proteins, in addition to sRAGE, as others have done previously [23]. The study by Liu and colleagues also showed that sRAGE levels in children doubled 1 h after snCPB; which is a similar fold increase to that reported in a pilot study in aduts undergoing snCPB [23] and also in our current study. Whilst associations with outcome were not reported in the previous study in adults [23], plasma sRAGE levels in children, immediately postoperatively, were shown to be an independent predictor of postop ALI [22]. However, it is difficult to draw comparisons between the lack of association between sRAGE levels and outcome we found since the patient groups are, as mentioned previously, very different. Generally, in critically ill adult patients raised plasma levels of sRAGE denote a worse outcome [15–18]. As the sRAGE levels measured in these studies are either similar or greater than the levels in the current study (1.93 ng/mL), differences in levels alone are unlikely to explain the lack of association of postoperative sRAGE and determinants of outcome. 

In contrast to the range of studies in which sRAGE or RAGE ligands have been measured after inflammatory insult, few can or have assessed preinsult levels. Another study showed that HMGB-1, a RAGE ligand, was a preoperative predictor of clinical course after surgery for thoracic oesophagectomy [34]. Thus, our findings that preoperative sRAGE levels are suggestive of an outcome are unique and intriguing. It is generally accepted that sRAGE acts as a competitive antagonist of cellular RAGE-mediated inflammation [1] by binding to circulating RAGE ligands it reduces ligand interaction with membrane-bound cellular RAGE. If sRAGE was to possess such anti-inflammatory actions, it might be expected to show an inverse, protective, association with duration of critical illness following snCPB as seen in studies of other conditions. However, an alternative hypothesis is that sRAGE could reflect ongoing low-level inflammation either cleaved from cells to reduce the consequences of membrane RAGE activation or secreted as a consequence of RAGE activation, as part of a positive feedback loop. Consistent with the two-hit hypothesis, higher preoperative sRAGE can be considered indicative of RAGE activation due to underlying conditions. It is of note that our previous findings in a similar patient cohort showed no significant difference in sRAGE levels with or without the following comorbidities: diabetes, pulmonary hypertension, stage of chronic kidney disease, and ischaemic heart disease (unpublished observation), suggesting it is unlikely that any of these comorbidities, at least alone, contributes to greater preoperative sRAGE levels. Surgical insult/CPB constitutes a second inflammatory hit (indicated by increased CRP and IL-8 levels in those patients in whom sufficient plasma sample was available for assay), which coupled with greater preoperative RAGE activation leads to worse outcome, that is, prolonged duration of care. 

A limitation to these investigations is the lack of standardisation of perioperative care. In common with the majority of similar observational cohort studies there was no standard protocol for specific management by the anaesthetist, perfusionist, or cardiac surgeon and thus, this might have varied slightly between patients. Also the anaesthetic, neuromuscular blocking agent and fluid resuscitation strategy all have the potential to modulate the inflammatory response and, potentially, postoperative sRAGE levels. A lack of information about all preoperative medications which might influence sRAGE levels is also a limitation of this study. Also, level 3 care is not by any means a perfect measure of “intensive” care but is potentially more sensitive than the length of time that a patient is located within an intensive care unit. Finally, focusing on a select group of patients (those at higher risk, i.e., with a higher EuroSCORE or who underwent more complex surgery) could, to some extent, limit the wider applicability of these findings. However, we chose to concentrate on this patient group in order to investigate our hypothesis; and having proven it, in principle, we would now propose to undertake a larger, inclusive study in order to further validate our findings. 

In conclusion, we have shown plasma levels of sRAGE prior to snCPB to be related to the requirement for extended (greater than median) duration of enhanced (i.e., level 3) critical care. No such association was shown with patient age, severity score for operative risk (EuroSCORE), or the duration of cardiopulmonary ischaemia, factors usually associated with morbidity and late mortality [35–37]. Preoperative sRAGE might be a biomarker that could offer superior risk stratification of patients undergoing major elective surgery and is worthy of further investigation as a potential therapeutic target.

## Figures and Tables

**Table 1:** The main clinical and biochemical characteristics, operative variables, and postoperative outcomes of study patients^a^.

Preoperative characteristics	
Age, median (IQR) years	71 (63–77)
Male, *n* (%)	81 (62.3)
Body mass index, median (IQR) kg/m^2^	25.7 (23.5–30.0)
Creatinine *μ*mol/L	91 (77.5–107.5)
EuroSCORE, median (IQR)	7 (4–8)

Operative variables	
Operation	
CABG alone, *n* (%)	1 (0.8)
Valve repair/replacement, *n* (%)	73 (56.2)
CABG plus valve surgery, *n* (%)	56 (43.1)
Cardiopulmonary bypass duration, median (IQR) min	115.5 (92.0–141.0)
Ischaemic time, median (IQR) min	85.5 (67.0–108.0)

Postoperative outcomes	
CRP postop day 1 versus preop, median (IQR) mg/L	61 (46–94) versus 2 (1–5)^†^
IL-8 2 h postop versus preop, median (IQR) pg/mL	47.6 (29.5–83.0) versus 3.7 (0.0–8.3)^‡^
PaO_2_ : FiO_2_ ratio, mean (SEM), mmHg	280.0 (8.3)
Incidence of ALI	0/130
Duration of ventilation, median (IQR) h	16.3 (11.5–21.8)
ICU length of stay, median (IQR) h	40.5 (21.7–49.9)
Level 3 care duration, median (IQR) h	19.3 (13.7–40.8)
Hospital length of stay, median (IQR) days	8.9 (7.2–13.1)
Hospital mortality, *n* (%)	5 (3.85)

IQR: interquartile range; SEM: standard error of the mean; ALI: acute lung injury; CABG: coronary artery bypass grafting; and ICU: intensive care unit.

^a^ *n* = 130 patients undergoing cardiac surgery necessitating cardiopulmonary bypass.

^†^ *P* < 0.001, *n* = 106. ^‡^ *P* < 0.001, *n* = 122. Wilcoxon matched pairs test.

**Table 2:** Binary logistic regression analysis of level 3 care with patient characteristics, operative variables,and postoperative outcomes^a^.

	Level 3 care > median (19.25 h)		Univariate analysis Odds ratio (95% CI)	*P*	Multivariate analysis Odds ratio (95% CI)	*P*
	No mean	Yes mean				
Age	67.6	70.3	1.0 (1.0-1.0)	0.165		
Ischaemic time	83.8	95.5	1.0 (1.0-1.0)	0.034^a^	1.01 (1.0-1.0)	0.887
CPB time	112.4	125.7	1.0 (1.0-1.0)	0.041^a^	1.0 (1.0-1.0)	0.550
Log creatinine	1.96	1.98	5.9 (0.3–124)	0.249		
Log BMI	1.42	1.42	1.1 (0.1–119)	0.9742		
Log EuroSCORE	0.73	0.77	2.2 (0.5–10.9)	0.325		
*Log sRAGE preop *	*−0.33 *	*−0.31 *	*8.5 (2.2–32.5) *	***0.002*** ^a^	*16.6 (1.8–155.4) *	***0.014*** ^b^
Log sRAGE postop	0.34	0.42	3.7 (1.0–14.5)	**0.065**	0.24 (0.0–2.7)	0.244
Log S100A8/9 preop	−0.04	0.13	1.5 (0.3–6.8)	0.617		
Log S100A8/9 postop	0.20	0.29	11.7 (1.5–89.4)	***0.018*** ^a^	5.7 (0.6–52.1)	0.120

CPB: cardiopulmonary bypass; BMI: body mass index; and *n* = 124.

^a^Significant relationship following univariate analysis.

^b^Significant relationship following multivariate analysis.

**Table 3:** Binary logistic regression analysis of hospital length of stay with patient characteristics, operative variables, and postoperative outcomes^a^.

	Hospital stay > median (8.94 days)		Univariate analysis Odds ratio (95% CI)	*P*	Multivariate analysis Odds ratio (95% CI)	*P*
	No mean	Yes mean				
*Age *	*66.7 *	*71.3 *	*1.0 (1.0-1.0) *	***0.016*** ^a^	*1.1 (1.0-1.1) *	***0.024*** ^b^
Ischaemic time	81.3	98.2	1.0 (1.0-1.0)	0.003^a^	1.0 (1.0-1.1)	0.100
CPB time	110.2	128.0	1.0 (1.0-1.0)	0.007^a^	1.0 (1.0-1.0)	0.422
Log Creatinine	2.0	2.0	8.0 (0.4–169.3)	0.182		
Log BMI	1.42	1.42	0.9 (0.0–102.1)	0.974		
Log EuroSCORE	0.74	0.76	1.5 (0.3–7.3)	0.609		
*Log sRAGE preop *	*−0.37 *	*−0.27 *	*18.3 (4.2–80.2) *	***0.000*** ^a^	*25.4 (1.3–483.6) *	***0.032*** ^b^
Log sRAGE postop	0.36	0.40	12.1 (2.7–54.2)	0.001^a^	2.9 (0.1–66.5)	0.500
Log S100A8/9 preop	−0.70	−0.15	4.6 (0.9–24.0)	**0.068**	2.4 (0.3–18.6)	0.407
Log S100A8/9 postop	0.16	0.32	3.7 (0.5–25.5)	0.181		

CPB: cardiopulmonary bypass; BMI: body mass index; and *n* = 124.

^a^Significant relationship following univariate analysis.

^b^Significant relationship following multivariate analysis.
